# Mosquito population structure, pathogen surveillance and insecticide resistance monitoring in urban regions of Crete, Greece

**DOI:** 10.1371/journal.pntd.0010186

**Published:** 2022-02-17

**Authors:** Emmanouil A. Fotakis, Konstantinos Mavridis, Anastasia Kampouraki, Sofia Balaska, Filianna Tanti, George Vlachos, Sandra Gewehr, Spiros Mourelatos, Antonios Papadakis, Maria Kavalou, Dimitrios Nikolakakis, Maria Moisaki, Nikolaos Kampanis, Manolis Loumpounis, John Vontas

**Affiliations:** 1 Institute of Molecular Biology and Biotechnology, Foundation for Research and Technology-Hellas, Heraklion, Greece; 2 Department of Crop Science, Pesticide Science Laboratory, Agricultural University of Athens, Athens, Greece; 3 Department Biology, University of Crete, Heraklion, Greece; 4 EcoDevelopment SA-Integrated Mosquito Control, Thessaloniki, Greece; 5 General Directorate of Public Health & Social Care of Region of Crete, Heraklion, Greece; USDA-ARS Center for Medical Agricultural and Veterinary Entomology, UNITED STATES

## Abstract

**Background:**

In Greece vector borne diseases (VBD) and foremost West Nile virus (WNV) pose an important threat to public health and the tourist industry, the primary sector of contribution to the national economy. The island of Crete, is one of Greece’s major tourist destinations receiving annually over 5 million tourists making regional VBD control both a public health and economic priority.

**Methodology:**

Under the auspices of the Region of Crete, a systematic integrative surveillance network targeting mosquitoes and associated pathogens was established in Crete for the years 2018–2020. Using conventional and molecular diagnostic tools we investigated the mosquito species composition and population dynamics, pathogen infection occurrences in vector populations and in sentinel chickens, and the insecticide resistance status of the major vector species.

**Principal findings:**

Important disease vectors were recorded across the island including *Culex pipiens*, *Aedes albopictus*, and *Anopheles superpictus*. Over 75% of the sampled specimens were collected in the western prefectures potentially attributed to the local precipitation patterns, with *Cx*. *pipiens* being the most dominant species. Although no pathogens (flaviviruses) were detected in the analysed mosquito specimens, chicken blood serum analyses recorded a 1.7% WNV antibody detection rate in the 2018 samples. Notably detection of the first WNV positive chicken preceded human WNV occurrence in the same region by approximately two weeks. The chitin synthase mutation I1043F (associated with high diflubenzuron resistance) was recorded at an 8% allelic frequency in Lasithi prefecture *Cx*. *pipiens* mosquitoes (sampled in 2020) for the first time in Greece. Markedly, *Cx*. *pipiens* populations in all four prefectures were found harboring the *kdr* mutations L1014F/C/S (associated with pyrethroid resistance) at a close to fixation rate, with mutation L1014C being the most commonly found allele (≥74% representation). Voltage gated sodium channel analyses in *Ae*. *albopictus* revealed the presence of the *kdr* mutations F1534C and I1532T (associated with putative mild pyrethroid resistance phenotypes) yet absence of V1016G. Allele F1534C was recorded in all prefectures (at an allelic frequency range of 25–46.6%) while I1532T was detected in populations from Chania, Rethymnon and Heraklion (at frequencies below 7.1%). Finally, no *kdr* mutations were detected in the *Anopheles* specimens included in the analyses.

**Conclusions/Significance:**

The findings of our study are of major concern for VBD control in Crete, highlighting (i) the necessity for establishing seasonal integrated entomological/pathogen surveillance programs, supporting the design of targeted vector control responses and; ii) the need for establishing appropriate insecticide resistance management programs ensuring the efficacy and sustainable use of DFB and pyrethroid based products in vector control.

## Author summary

The island of Crete, is one of Greece’s major tourist destinations, receiving over 5 million tourists annually, making control of vector borne diseases (VBD) like West Nile virus (WNV) both a public health and economic priority. A comprehensive surveillance network targeting mosquitoes and associated pathogens was established in Crete for the years 2018–2020. Important disease vectors (*Culex pipiens*, *Aedes albopictus* and *Anopheles superpictus*) were recorded across the island. The presence of WNV antibodies in sentinel chickens was also recorded and, notably, preceded human WNV occurrence in the same region by approximately two weeks. Mutations associated with resistance to insecticides used for vector control were also detected; most importantly the chitin synthase mutation I1043F (associated with high diflubenzuron resistance) was recorded for the first time in Greece. The findings of our study are of major concern for VBD control in Greece (and Crete specifically), highlighting (i) the necessity for establishing seasonal surveillance programs and ii) the need for establishing appropriate insecticide resistance management programs for sustainable vector control.

## Introduction

As of the late 2000s, southern European countries have witnessed the emergence and resurgence of important vector borne diseases (VBDs). Within this changing epidemiological scene, between 2010 and 2020 Greece has faced 9 (annual) West Nile virus (WNV) outbreaks [1] and a documented total of 110 locally-acquired malaria cases [2] collectively threatening public health and the tourist industry, a major pillar of the Greek economy accounting for 20.8% of the country’s gross domestic product [3,4]. The island of Crete, one of Greece’s major tourist destinations, receives more than 5 million tourists every year, generating over 3 billion euros in revenue [5]; making VBD control both a public health and regional/national economic priority.

Notably, southern Europe displays a rich mosquito fauna comprised of multiple vector species, many of which are also present in Greece. Prominent disease vectors recorded in the country include (i) the WNV vectors *Culex modestus* and *Culex pipiens* s.s. [6,7] which consists of three biotypes: *Cx*. *pipiens pipiens*, *Cx*. *pipiens molestus*, and a hybrid form considered an important bridge vector due to its opportunistic feeding behaviour [8]; (ii) the major arboviral vector *Aedes albopictus* [9,10] and; (iii) the malaria vectors: *Anopheles sacharovi* and *Anopheles superpictus;* as well as the secondary/suspected malaria vectors: *Anopheles maculipennis* s.s., *Anopheles melanoon*, *Anopheles algeriensis*, *Anopheles claviger*, *Anopheles hyrcanus*, and *Anopheles plumbeus* [6,7,11–13].

Of the aforementioned epidemiologically relevant species; *Cx*. *pipiens*, *Ae*. *albopictus*, *An*. *claviger*, and *An*. *sacharovi* have also been reported from Crete in addition to the nuisance species *Culex territans*, *Aedes caspius*, *Aedes cretinus*, *Aedes detritus*, *Aedes dorsalis*, *Culiseta longiareolata*, *Culiseta annulata*, and *Culiseta sabochrea* [12,14]. However, robust information on the species composition, abundance and spatiotemporal dynamics across the island remains scarce.

Concurrent to the dominant and widespread distribution of the major WNV vector *Cx*. *pipiens* s.s. in southern Europe, WNV is currently the leading cause of VBD in the region, with Greece carrying a high disease burden [15]. Between 2010–2020 a total of 1360 human WNV cases and 192 associated deaths were recorded from Greece [16,17], while in 2019 the 227 Greek WNV cases accounted for more than half of the overall cases reported in the European Union [18].

As WNV amplifies through an enzootic cycle between birds and mosquitoes, with humans and other mammals constituting incidental and “dead-end” hosts [19], an essential prerequisite for alleviating the risk of transmission to humans is the establishment of comprehensive WNV surveillance schemes to organize evidence based control. Several European countries devote significant resources annually to WNV surveillance including: surveillance of (i.e., post mortem examination of dead organisms manifesting signs of WNV infection), and/or active surveillance (i.e., mosquito, sentinel bird, and sentinel equid screening)[20].

Active surveillance has proven highly advantageous and of great operational relevance as the detection of WNV in mosquitoes and seroconversion in live chickens or horses usually precedes the appearance of dead animals and human cases, serving as an early warning system component [21,22]. Active WNV surveillance programs have been deployed in the past in northern and central Greece [21–24] yet no such systematic monitoring schemes have been implemented in Crete despite the occurrence of three human WNV clinical cases between 2017–2018 in the prefecture of Rethymnon and the detection of positive equid seroconversions in 2017 in the prefectures of Chania, Heraklion, and Lasithi [25].

The re-appearance of autochthonous *Plasmodium vivax* malaria cases in several foci in Greece following disease eradication in the 1970s [26] alongside the annual occurrence of imported malaria cases (including reports from Crete) [27] poses an added VBD public health threat, in turn highlighting the need for plasmodium monitoring in local vector populations in support of minimizing the risk of malaria transmission.

In the absence of protective human vaccines against most VBD and a number of limitations accompanying therapeutic drugs, disease prevention and control largely depend on controlling vector populations through the use of insecticides [28]. The larvicides *Bacillus thuringiensis israelensis* (*Bti*), diflubenzuron (DFB), and pyrethroid based adulticides comprise the main insecticides used for vector control in the European Union (EU) [29] and are applied primarily through large-scale municipality/prefecture level control programs. The same insecticides/active ingredients are used against a number of agricultural pests [30].

In Greece *Bti* and DFB applications compose the majority of vector control interventions, while pyrethroid insecticide formulations are principally used in outbreak situations and/or settings of increased epidemiological relevance [31]. In Crete, local mosquito control programs mainly rely on DFB whereas pyrethroid based products are applied extensively against the olive fruit fly, *Bactrocera oleae* (a major agricultural pest in the region) [32]. *Bti*, which exhibits a lower risk of resistance, is used to a lesser extent for mosquito control purposes and primarily against agricultural pests in organic farm/cultivation settings.

An emerging problem in southern Europe associated with extensive and repeated use of available insecticides in vector and agricultural pest control programs is insecticide resistance, impeding the effectiveness of control efforts. Insecticide resistance may be conferred through behavioral changes, cuticular modifications, target site insensitivity, and increased metabolic detoxification with the latter two mechanisms comprising the most well studied and documented resistance traits [33].

Alarmingly, high DFB resistance attributed to the chitin synthase (*CHS*) mutations I1043L, I1043M, I1043F was recently recorded in *Cx*. *pipiens* populations from northern Italy [34–36] and western Turkey (with resistant allele frequencies reaching > 50%) [37], however no mutations have been detected to date in *Cx*. *pipiens* or *Ae*. *albopictus* populations from Greece [9,35].

In addition, the voltage gated sodium channel (VGSC) knock-down resistance (*kdr*) mutations: L1014F (recorded in multiple *Anopheles* and *Culex* species and upon homozygosity associated with significant pyrethroid resistant phenotypes, especially when in combination with P450 metabolic resistance) [38,39]; L1014C (recorded in *Anopheles* and recently *Culex* mosquitoes and shown through in vitro functional evidence to confer a slight VGSC sensitivity reduction against permethrin and deltamethrin [40]); F1534S/L/C and I1532T (which upon their detection in *Aedes* vector species, have been related to a VGSC loss of sensitivity against type I pyrethroids [41]); and V1016G (also occurring in Aedes mosquitoes and correlated to a loss of sensitivity against both type I and type II pyrethroid insecticides [42]) have been reported respectively in several Culex and Aedes populations from Greece and neighbouring countries (little data is currently available on European Anopheles populations).

Resistance to acetylcholinesterase (AChE) agonists, organophosphates (OPs), and carbamates has rarely been analyzed molecularly in European vector populations. However, *Ace-1* target-site mutations and overexpression of the carboxylesterases (CCEs) *CCEae3a* and *CCEae6a* has been documented in *Cx*. *pipiens* and *Ae*. *albopictus* from Greece [6,9,43].

Complementary bioassay evidence from Greece includes reports on suspected deltamethrin (pyrethroid) resistance in *Ae*. *caspius* and *An*. *hyrcanus* populations from northern Greece [6]. Deltamethrin and temephos (organophosphate) resistance in *Cx*. *pipiens* from northern and central Greece [6,43]. and malathion (organophosphate) resistance from *Ae*. *albopictus* populations, including a population from Heraklion, Crete [9].

Integrative surveillance systems that encompass both an entomological and epidemiological component are a core requirement for effective VBD control/prevention programs [44]. Specifically, generating information on the mosquito fauna composition, vector population spatiotemporal dynamics, vector insecticide resistance status against major insecticides and the seasonal presence/circulation of mosquito-borne pathogens in vectors and reservoir/dead end hosts provides essential information for assessing the VBD scene and guiding the deployment of evidence based vector/disease control interventions in a targeted, cost effective and timely manner [45].

Despite the VBD history in Greece, the currently available information and systematic generation of entomological, insecticide resistance, and epidemiological data in certain regions, including the island of Crete, is poor and fragmented. Under the auspices of the Region of Crete, a systematic integrative surveillance network targeting mosquitoes and their associated pathogens was established in Crete between 2018–2020, in light of supporting evidence based regional vector control programs. The main actions presented here include: 1) monitoring the species composition and population dynamics in the four prefectures of Crete with a special focus on the island’s northern coastline; 2) recording the presence and intensity of flavivirus infections in mosquito populations and WNV in sentinel chickens and; 3) analyzing the vector populations’ insecticide resistance status against major insecticides used for vector control in Crete/Greece.

## Materials & methods

### Study region, mosquito surveillance and sample handling

**Fig 1:**
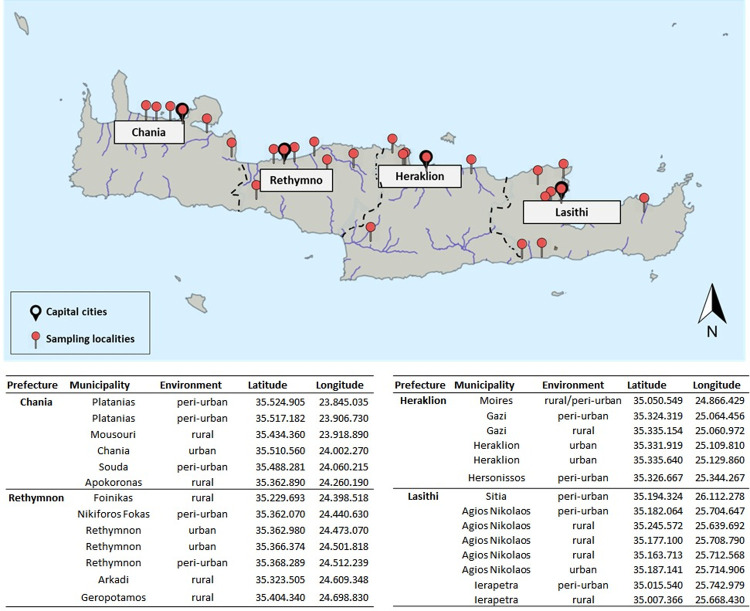
Study region (island of Crete) and adult mosquito sampling locations (red pins). The base layer of the map was obtained from http://geodata.gov.gr/ (an open data source accessible to everyone) and is made available under licence CC BY 3.0l.

Between 2018–2020, Crete, (Greece) established an integrated mosquito surveillance program. Crete, is the largest (8,260 km^2^), and most populated (623,000 residents) Greek island [46]. It is the southernmost administrative district of Greece, and comprises of four prefectures: (from West to East) Chania, Rethymnon, Heraklion, and Lasithi **(Fig 1)**.

Major towns (where over 70% of the island’s population reside), and tourist accommodations are located on the North coast [47]. The rest of the population is scattered amongst villages throughout the remainder of the island. Agricultural activity is intense in all prefectures with cropland covering 37.7% of the island’s total land area [48].

Entomological surveillance was conducted from June to December in 2018, and from April to October in 2019 and 2020. The sampling locations were selected based on a combination of factors prioritized per: a) the island’s major tourist destination and accommodation sites, b) the island’s population layout, c) areas with intense mosquito nuisance problems, d) historical records of VBD occurrences in vertebrate hosts and e) previous knowledge on proliferative mosquito breeding sites. Center for Disease Control (CDC)-type light traps supplied with 1.5 kg CO2 were deployed every two weeks (from 18.00 pm to 8.00 am / per sampling event) in fixed sampling locations within each prefecture (i.e. 6 sampling stations in Chania, Heraklion, 7 in Rethymnon, and 8 in Lasithi) for collecting adult mosquitoes. Supplementary collections were carried out in response to generated data indicating VBD pathogen presence or transmission **(Fig 1)**.

All adult collections were immediately stored at -20°C and transferred to the laboratory. Female specimens were identified to species morphologically [13,28] (under cold chain conditions) and stored in 70% ethanol (for inclusion in molecular diagnostic assays, e.g., species identification and resistance mutations and *Plasmodium* parasite detection), or at -80°C (for Flavivirus detection). Prior deep freezing (-80°C) the legs of the respective specimens were detached from the main body and stored in 70% ethanol—for species identification. A total of 967 sampling events (s.e) were conducted during the program, with 227 s.e in Chania, 227 s.e in Heraklion, 227 s.e in Lasithi, and 286 s.e in Rethymnon.

### Genomic DNA and RNA extraction

Genomic DNA (gDNA) was extracted from single adult female mosquitoes using the DNAzol reagent (Invitrogen, Carlsbad, CA, USA) according to manufacturer’s instructions. Total RNA was isolated from extraction pools of *Cx*. *pipiens* or *Ae*. *albopictus* mosquitoes using the TRI reagent protocol (Invitrogen, Carlsbad, CA, USA) following the manufacturer’s instructions.

Each extraction pool was comprised of up to 30 mosquitoes of the same species, collected from the same or neighbouring municipalities and from the same collection date. The concentration and purity of the extracted (pooled) RNA was determined by spectrophotometry using a NanoDrop 2000c spectrophotometer (Thermo Fisher Scientific, Waltham, MA, US) and its integrity was confirmed via 1% w/v agarose gel electrophoresis as previously described [49].

### Mosquito species molecular identification

Molecular species identification analyses was conducted in order to investigate, verify and monitor the presence, abundancy and geographical distribution of vector species. Species identification was based on the PCR amplification KAPA Taq PCR Kit (KAPA Biosystems) of specific molecular markers. All assays (i.e., primers, reactions, thermal protocols and product sizes) are described in detail in **(S1 and S2 Tables).** Members of the *Culex pipiens* complex (*Cx*. *pipiens*, *Cx quinquefasciatus*, *Cx*. *pallens*, *Cx*. *australicus*, and *Cx*. *torrentium*) were distinguished based on polymorphisms in the intron region of acetylcholinesterase-2 gene (*ace-2*) and the use of species specific primers [50]. Specimens identified as *Cx*. *pipiens* were further analyzed to biotype (*Cx*. *pipiens pipiens*, *Cx*. *pipiens molestus*, Cx. *pipiens* hybrid) relying on polymorphisms in the 5’ flanking region of the microsatellite locus CQ11 [51]. *Ae*. *albopictus* was discriminated from *Ae*. *aegypti* and the non-vector species *Ae*. *cretinus* by amplification of the internal transcribed spacer two gene (ITS2) and the different size diagnostic fragments produced for each species as described in [52].

*Anopheles* mosquitoes were molecularly differentiated by partial amplification of the ITS2 and mitochondrial cytochrome c oxidase I gene (COI) [53,54]. A small amount of the respective PCR products (5 μl) were electrophoresed on a 1% w/v agarose gel to verify the presence of the correct size amplicon [53,54]. The remaining amount from each reaction was purified using the Nucleospin PCR & Gel Clean-Up Kit (Macherey Nagel) and sequenced (with the primer 5.8S for ITS2 and C1-J-1718 for COI) using the Sanger method (CeMIA S.A., Larissa, Greece) [53,54]. All generated sequences were analyzed using the NCBI BLAST algorithm and the sequence alignment editor BioEdit 7.2.5 (https://bioedit.software.informer.com/7.2/).

### Flavivirus detection in mosquitoes

A conventional one-step Reverse Transcriptase-PCR (RT-PCR) assay was performed to assess the presence of Flaviviruses in *Ae*. *albopictus* and *Cx*. *pipiens* pools, based on the method described by [49] **(S1 and S2 Tables)**. WNV-specific detection analysis was performed in all pools (regardless of the respective Pan-Flavivirus assay outcome) with a multiplex Real Time one step RT-PCR TaqMan assay that simultaneously detects and differentiates WNV-lineage 1 from WNV-lineage 2 [55]. Samples were amplified in triplicate and each run always included a non-template control and a positive control supplied by BEI resources (Manassas, VA, US) (Genomic RNA from West Nile Virus, Bird 114, Catalog No. NR-9573).

### WNV Antibody detection in sentinel chickens

WNV epidemiological surveillance was performed via monitoring the presence of antibodies against WNV in the blood sera of backyard sentinel chickens. Blood collections were conducted between August–September in 2018, and July-August in 2019 and 2020 from a total of 588 chickens (sampling events) aged 5.81 ± 0.20 months.

Blood from chickens was collected in 1.5 ml microcentrifuge tubes, allowed to clot for 10 min and transferred to the laboratory on ice. Samples were then centrifuged immediately (3,000×g, 10 min, 4°C) and sera were transferred to clear tubes and stored at -20°C until analysis. Sera were tested for the presence of antibodies against the WNV envelope protein (E), using a competitive enzyme-linked immunosorbent assay (cELISA) kit available commercially (ID Screen West Nile Competition; ID.Vet Innovative Diagnostics, Montpellier, France), according to the manufacturer’s instructions.

### Monitoring of target site resistance mutations

The presence of important insecticide resistance markers was investigated using PCR assays (KAPA Taq PCR Kit; KAPA Biosystems, Wilmington, MA, US) which are described in detail in **(S1 and S2 Tables)**. All assay amplicons were purified or extracted from agarose gels using the Nucleospin PCR & Gel Clean-Up Kit (Macherey Nagel) and sequenced with the Sanger method (CeMIA S.A., Larissa, Greece). Sequences were analyzed using the sequence alignment editor BioEdit 7.2.5.

*Culex* mosquitoes were individually genotyped for the presence of the *kdr* mutations L1014F/C/S on the voltage-gated sodium channel gene (*VGSC*) using the outer primers and PCR conditions described in [56]. Five μl of each PCR reaction was visualized on an agarose gel and the remaining product was purified and sequenced, using the primer Cx1014F. *Anopheles* specimens were also genotyped for the *kdr* mutations L1014F/C/S based on the protocol described in [57]. The purified PCR products were sequenced using the primer AnHR [57].

In *Ae*. *albopictus*, monitoring of the *kdr* mutations V1016G (VGSC domain II) and I1532T, F1534C/L/S (VGSC domain III) was conducted via PCR amplification of the respective domain regions encompassing the mutation sites. In particular, following individual specimen DNA extraction, the gDNA from 5 to 8 *Ae*. *albopictus* specimens was pooled and used as a template for the amplification of *VGSC* domain II. The PCR products were purified and sequenced with the primer kdr2F, as described in [58]. The *VGSC* domain III fragments were amplified separately in each specimen. The generated amplicons were visualized on agarose gels, extracted and sequenced using the primer aegSCR8 [58].

*Cx*. *pipiens* and *Ae*. *albopictus* specimens were also screened for the presence of the mutations I1043L/M/F in the chitin synthase-1 gene (*CHS-1*). Following DNA extraction at the individual level, a fragment of the *CHS-1* gene spanning the locus 1043 was amplified in species specific pooled gDNA templates (comprising of 5 to 8 specimens each). Upon positive mutation detection the respective pool’s samples were analyzed individually. The produced amplicons were sequenced with primers Kkv F1 (*Cx*. *pipiens*) and KkvF3 (*Ae*. *albopictus*)[9,34].

## Results

### Genus/Species composition and population dynamics in urban regions of Crete

**Fig 2:**
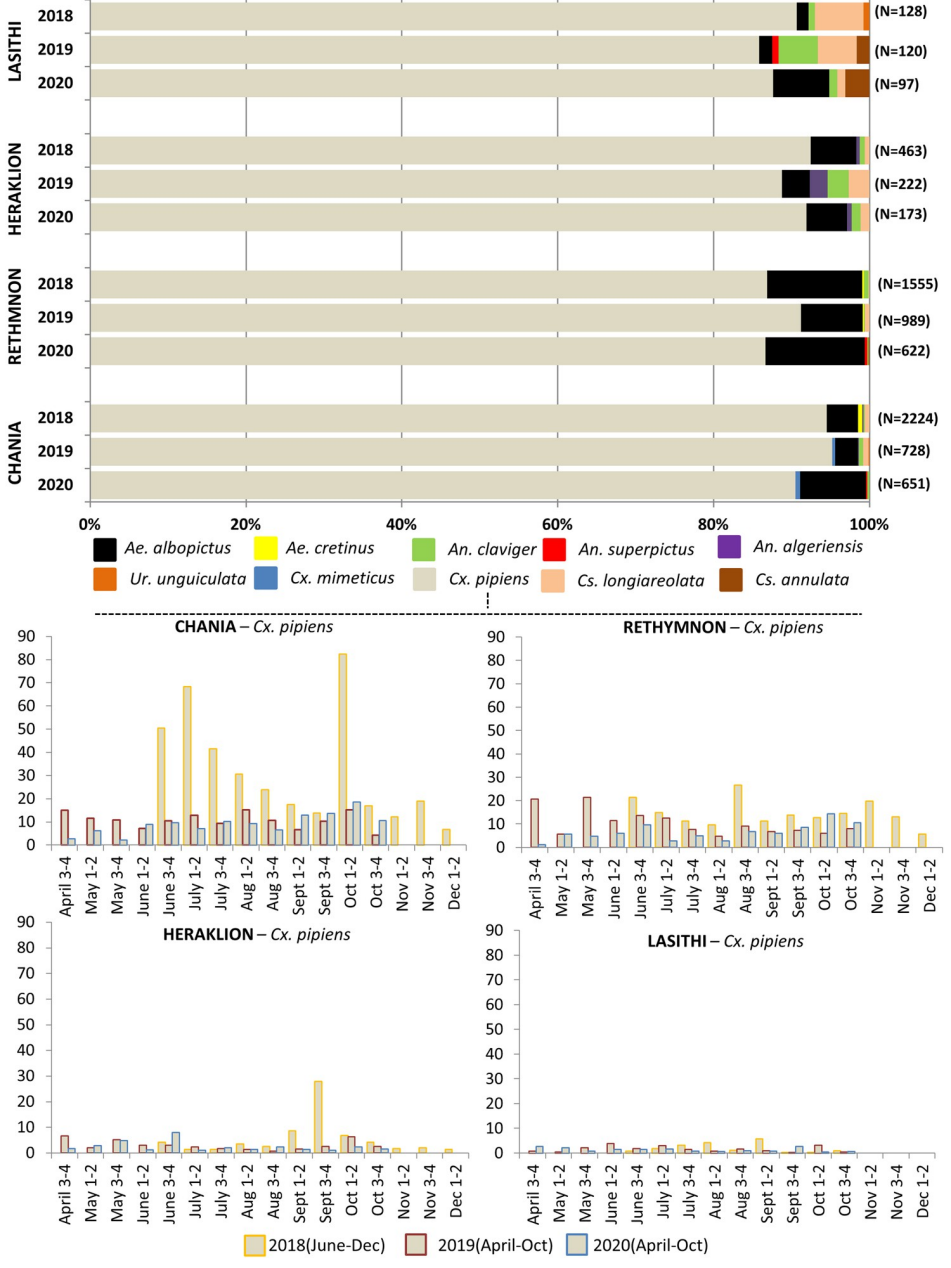
Adult female mosquito species composition per prefecture (top) and prefecture level Culex pipiens ss population dynamics (bottom). N: total number of female adult mosquitoes collected per prefecture per surveillance year. Each column (bottom) corresponds to the mean of the samples collected per trap per night. (1–2);(3–4): collections corresponding to the first and last two weeks of the month respectively. (June-Dec): mosquitoes were collected from June to December. (April-Oct): mosquitoes were collected from April to October. All data correspond to collections conducted with CDC light traps baited with dry ice (potentially under-recording the presence/abundance of certain mosquito species).

A total of 7992 adult mosquitoes were collected in the region of Crete during the years 2018–2020 (i.e. 967 sampling events) with 84.9% of sampled specimens (N = 6789) collected in the western prefectures (i.e Chania and Rethymnon). Mosquitoes of the *Culex* genus were the most abundant, representing over 85% of the respective mosquito catches within each prefecture/surveillance year, followed by *Aedes*, *Culiseta*, *Anopheles*, and *Uranotaenia* genera **(Fig 2)**. Notably all *Culex* mosquitoes (i.e N = 6810 specimen, apart from N = 7 identified as *Culex mimeticus*) were morphologically identified as members of the *Culex pipiens* complex. Of the N = 53 *Culiseta* samples, N = 48 were identified as *Culiseta longiareolata* and N = 5 as *Culiseta annulata* while the few *Uranotaenia* collected (N = 4) were classified as *Uranotaenia unguiculata*. Within the *Aedes* genus, the majority of specimens (N = 567) were *Ae albopictus*, while a small number of samples were identified as *Ae*. *cretinus* (N = 18) and *Ae*. *detritus* (N = 4). Amongst the anopheline mosquitoes 3 species were identified morphologically; *An*. *claviger* (N = 40), *An*. *algeriensis* (N = 12) and *An*. *superpictus* (N = 4).

The overall (3 year) *Cx*. *pipiens* population densities were highest in Chania (N2018-20 = 3407) followed by Rethymnon (N2018-20 = 2789), Heraklion (N2018-20 = 784), and Lasithi (N2018-20 = 304) **(Fig 2)**. The island’s annual (cumulative) *Cx*. *pipiens* numbers peaked in 2018 followed by a 52.7% population size reduction in 2019 and 38.1% reduction in 2020 (in reference to 2019). The population drop was distinctly evident in Chania (reportedly the prefecture with the highest *Culex* numbers): the seasonal average (s.a) of *Cx*. *pipiens* collected per trap per night in Chania in 2018 (s.a = 31.95 ± 6.9) dropped three fold in 2019 (s.a = 10.7± 0.94) (P = 0.011), which was further slightly, but not statistically significantly reduced (s.a = 9.12± 1.2) in 2020 (P > 0.05).

As displayed in (**Fig 2)** the 2018 Chania *Cx*. *pipiens* populations showed high activity in the months June, July followed by a population decrease in August and September, and an increase in early October where the population activity peaked. Populations then decreased through November into December. In 2019 and 2020 the Chania *Cx*. *pipiens* populations were collected at a steady rate, dropping in late October. A similar steady trend was observed in the *Culex* populations from Rethymnon, Heraklion, and Lasithi throughout all surveillance years with the exception of a population peak in Heraklion in late September of 2018.

All *Cx*. *pipiens* complex mosquitoes included in the molecular analyses (N = 401 specimens) were identified as the major WNV vector *Culex pipiens* s.s. **(Table 1)**. Dowstream biotype analysis in a subset (N = 149) of the *Cx*. *pipiens* s.s specimens, reported the presence of all three biotypes (i.e., pipiens molestus, pipiens/molestus hybrid forms) in the four prefectures. The *Cx*. *pipiens* pipiens/molestus hybrid form, which is considered a prominent WNV vector, was recorded at frequencies ranging from 20.5% to 41.7% in the prefectures of Chania, Rethymnon, and Heraklion **(Table 1)**.

**Table 1:** Vector species population structure.

Prefecture	*Culex*						*Aedes*			*Anopheles*			
	N	*Cx*. *pipiens* ss	N	*Cx pipiens* ss biotype			*N*	*Ae*. *albopictus*	*Ae*. *cretinus*	*N*	*An*. *claviger*	*An*. *algeriensis*	*An*. *superpictus*
				molestus	pipiens	hybrid							
Chania	115	100(%)	49	28.6(%)	45(%)	26.4(%)	89	86.5(%)	13.5(%)	9	66.7(%)	22.2(%)	11.1(%)
Rethymnon	122	100(%)	39	18(%)	61.5(%)	20.5(%)	92	97.8(%)	2.2(%)	4	50(%)	-	50(%)
Heraklion	101	100(%)	36	19.4(%)	38.9(%)	41.7(%)	7	100(%)	-	6	50(%)	50(%)	-
Lasithi	63	100(%)	25	24(%)	72(%)	4(%)	6	100(%)	-	10	90(%)	-	10(%)

**Fig 3:**
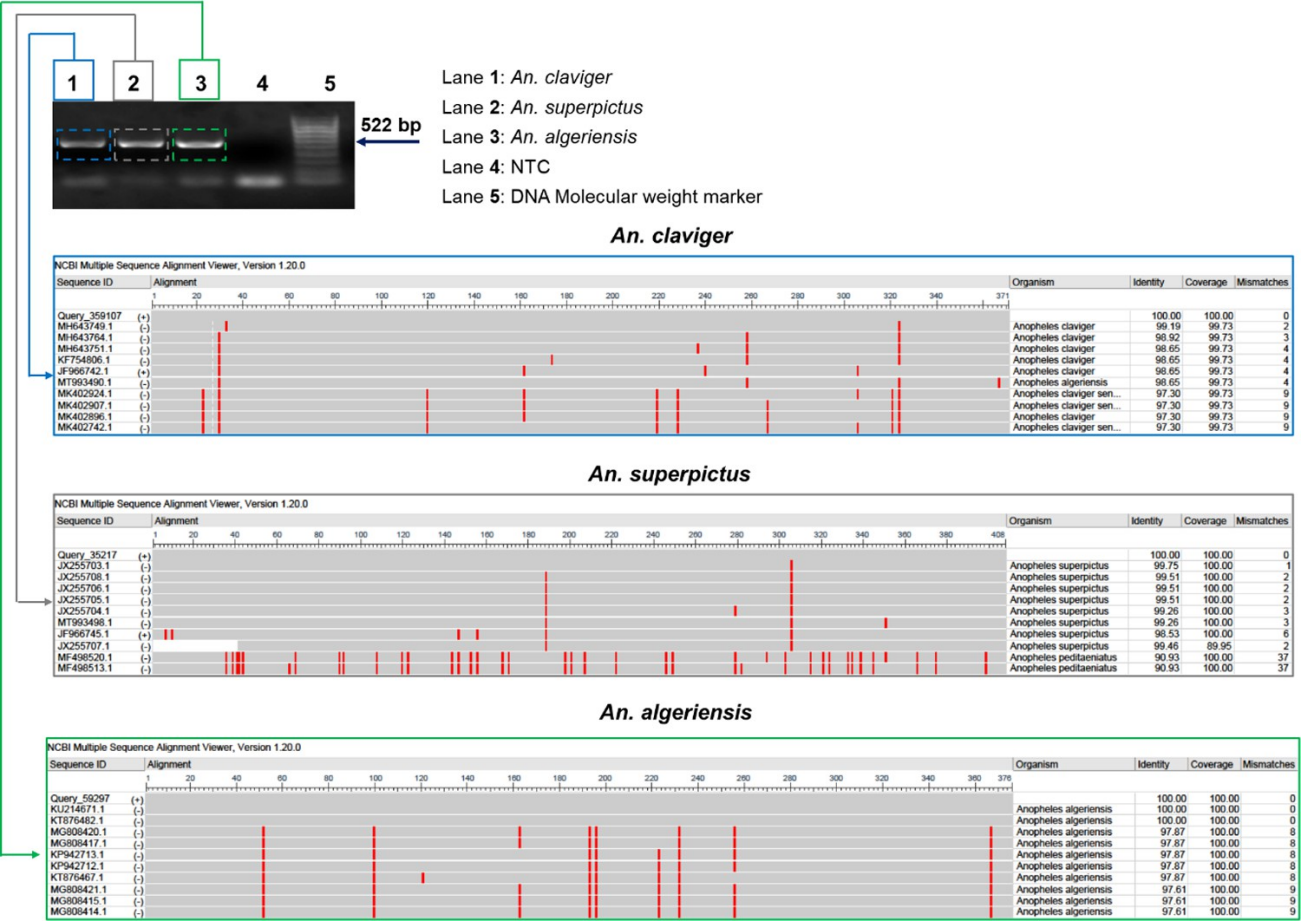
Detection of the malaria vector *An*. *superpictus*. Application of COI assay in *Anopheles* specimens with regular PCR (top), Megablast analyses (showing the 10 first alignments) for species discrimination (bottom).

*Aedes* specimen analysis (in N = 194 samples) revealed >86% representation within the *Aedes* genus samples/in each prefecture for*Ae*. *albopictus* over the morphologically similar *Ae*. *cretinus*, reported only in Chania and Rethymnon, at lower numbers **(Table 1)**. *Anopheles* species discrimination (in N = 29 specimen) recorded the malaria vector *Anopheles superpictus* (**Fig 3**) in Chania, Rethymnon and Lasithi, the suspected/secondary malaria vector *Anopheles claviger* in all prefectures and the suspected malaria vector *Anopheles algeriensis* in Chania and Heraklion **(Table 1)**.

### Pathogen/infection monitoring in vector species and sentinel chickens

**Fig 4:**
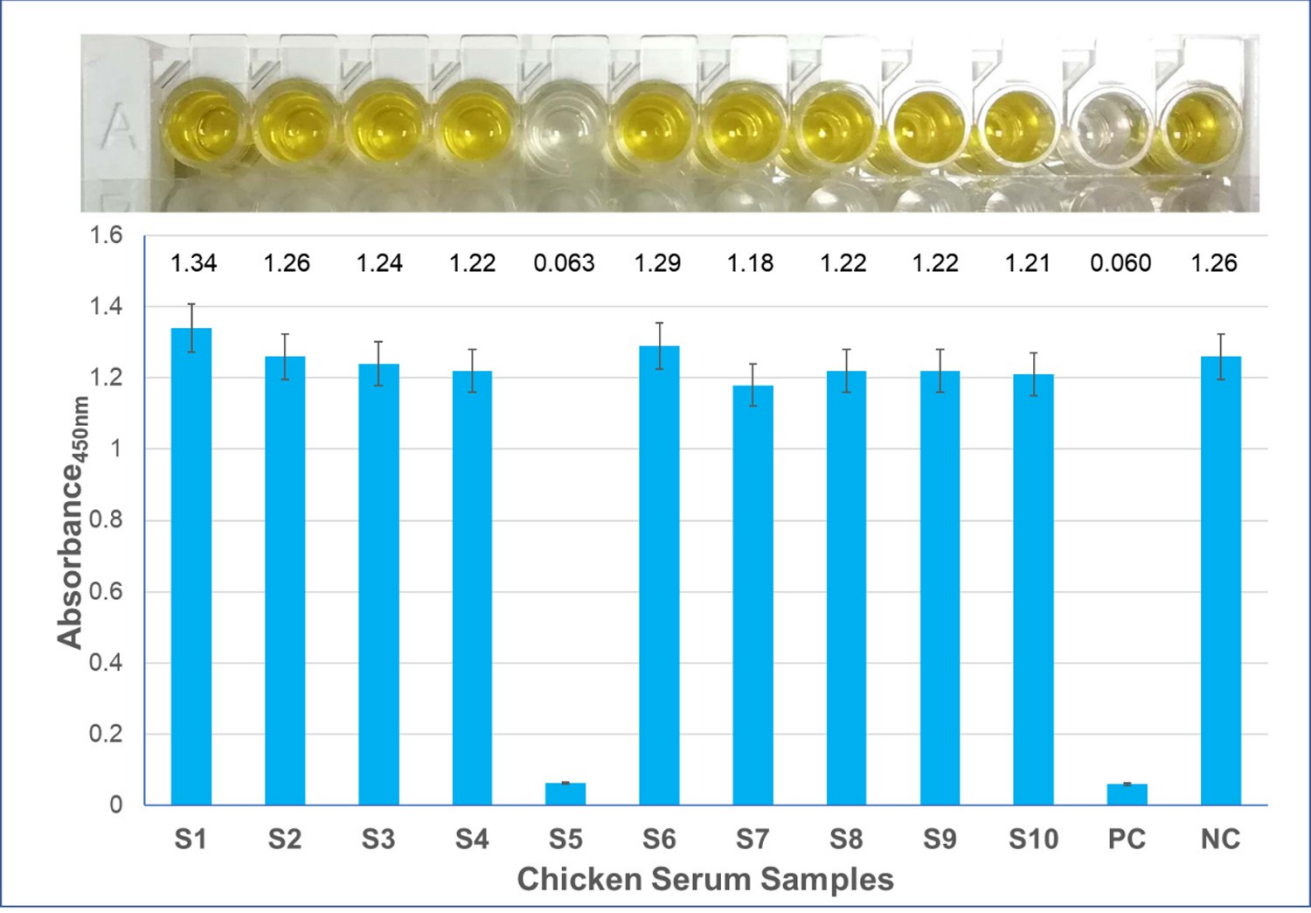
Application of the competitive ELISA assay for the detection of WNV anti-pr-E antibodies in 10 chicken sera from Crete (samples S1-S10, 2018). PC: positive control, NC: negative control. Absorbance (Abs) values in 450 nm are given in the parentheses. The % Signal to Noise (%S/N) is calculated as: %S/N = (Abs_sample/Abs_negative) x 100%. The result is considered positive if %S/N ≤ 40.0%, doubtful if %S/N falls between 40.0%-50.0% and negative if %S/N > 50.0% Sample S5 is positive (%S/N = 5.0%), whereas the remaining samples are negative.

During the surveillance program a total of N = 590 sentinel chicken blood serum samples were analysed from all prefectures for the presence of antibodies against WNV. WNV specific antibodies were detected in 4 of the 235 chicken samples analysed in 2018, corresponding to a positive antibody rate of 1.7% (for the Crete 2018 samples) (**Fig 4**). All positive samples were from Rethymnon prefecture.

Regarding pathogen monitoring in vector populations, none of the pooled results from *Cx*. *pipiens (*NCx.pipiens pools = 239 / NCx..pipiens specimens = 3031) or *Ae*. *albopictus* (NAe. Albopictus pools = 18 / NAe. albopictus specimens = 191) analyzed for Flavivirus/WNV infections, were found positive (**S1 Fig and S3 Table**). Likewise, all *Anopheles* samples analysed (n = 25) were negative for *P*. *vivax* and *P*. *falciparum* infections.

### Vector insecticide resistance

*Cx*. *pipiens* specimens (N = 253) sampled from all prefectures between 2018–2020 were analysed for the presence of DFB resistance mutations at the CHS loci 1043. The mutation I1043F, associated with high DFB resistance, was recorded as homozygous in two samples collected from Lasithi (Agios Nikolaos) in 2020, corresponding to an 8% allelic frequency (in the 2020 Lasithi population). All 2018–2020 samples from Chania, Rethymnon, and Heraklion were homozygous for the wild type-susceptible allele I1043 **(Table 2)**. Genotyping of the CHS locus 1043 in *Ae*. *albopictus* (N = 115 samples) did not detect any of the I1043L/M/F mutations linked to DFB resistance.

**Table 2:** Allele frequencies (%) of VGSC loci [1014, 1016, 1532, 1534], CHS locus [1043] and genotypic frequencies (%) of [CCEae3a, CCEae6a] (1 vs multiple gene copies).

Associated Insecticide(s)	Species / loci	Collection years	Prefecture	N	Allelic/genotypic frequencies % ^v^ (min-max frequency values %^)							
**Pyrethroids**	***Cx*. *pipiens* VGSC locus 1014**				WT allele				*kdr* mutations			
					**L1014**				**1014F**	**1014C**		**1014S**
		2018-2019-2020	Chania	108	7.9 (0–12)				16.7 (12.9–26.5)	74 (62–78.8)		1.4 (0–6)
			Rethymnon	102	6.4 (0–8.9)				15.8 (6.7–19.4)	74.3 (66.1–93.3)		3.5 (0–5.6)
			Heraklion	90	4.4 (2–10)				15 (9–23.3)	80 (66–89)		0.6 (0.2)
			Lasithi	63	4.8 (0–12.5)				19.8 (11.8–30)	75.4 (57.5–85.3)		0
	***Ae*. *albopictus* VGSC loci 1016, 1532, 1534**				WT alleles				*kdr* mutations			
		2018-2019-2020			**V1016**	**I1532**		**F1534**	**1016G**	**1532T**		**1534C**
			Chania	51	100	95.7 (93–100)		66.3 (56.7–70.2)	0	4.3 (0–6.3)		33.7 (30–43)
			Rethymnon	44	100	97.7 (96–98.1)		53.4 (51–57.1)	0	2.3 (1.5–3.6)		46.6 (42.9–49)
			Heraklion^#^	7	100	92.9		64.3	0	7.1		35.7
			Lasithi^#^	4	100	100		75	0	0		25
**DFB**	***Cx*. *pipiens* CHS1 locus 1043**				WT allele				*CHS1* mutations			
		2018-2019-2020			**I1043**				**1043M**	**1043F**		**1043L**
			Chania	59	100				0	0		0
			Rethymnon	87	100				0	0		0
			Heraklion	50	100				0	0		0
			Lasithi	52	96.16 (0–92)				0	3.84 (0–8)		0
**Ops** ^**!**^	***Ae*. *albopictus* CCEae3a CCEae6a loci**				WT genotypes				CCEae3a/CCEae6a amplicons			
					**CCEae3a (1 gene copy)**		**CCEae6a (1 gene copy)**		**CCEae3a (≥ 2 copies)**		**CCEae6a (≥ 2 copies)**	
		2018	Chania	19	73.7		73.7		26.3		26.3	
			Rethymnon	23	52.2		61		47.8		39	
		2019	Heraklion	19	68.4		73.7		31.6		26.3	
		2018	Lasithi	2	100		100		0		0	

**N** = number of samples

**^v^**the relative allelic frequencies derived from all the analysed 2018–20 (grouped) specimens/per prefecture

**^**the minimum and maximum relative allelic frequency values recorded (per prefecture) from annually grouped specimens (i.e min-max annual values for the period 2018–2020); **WT**: wild type (susceptible) allele(s)

^**#**^ min-max frequency values are not given (only samples from 2020 were analysed); **DFB**: diflubenzuron; **Ops**: organophosphates

^**!**^Analyses were conducted by [9] following independent sample collections.

*Cx*. *pipiens* mosquitoes (N = 362) were also analyzed for the presence of pyrethroid resistance mutations at the VGSC loci 1014. Three mutations—L1014F/C/S were recorded with the wild type/susceptible allele L1014 reporting frequencies (ranging from 4.4 to 7.9%) in the four prefectures **(Table 2)**. Mutation L1014C was detected at frequencies ≥ 74% in all prefectures, followed by mutation L1014F with frequencies ranging from 15–19.8%. Mutation L1014S was recorded in Chania, Rethymnon and Heraklion, at frequencies ranging from 0.6–3.5% **(Table 2)**.

*Kdr* mutation analyses in *Ae*. *albopictus* (N = 106) recorded mutations in both Vgsc domain III loci genotyped (i.e., I1532 and F1534). Mutation I1532T was recorded from Chania (4.3%), Rethymnon (2.3%), and Heraklion (7.1%); and mutation F1534C was detected in all prefectures Specifically from Rethymnon at a 46.6% frequency, Heraklion (35.7%), Chania (33.7%) and Lasithi (25%). Domain II loci 1016 analyses recorded only the wild type/susceptible allele V1016 in all (N = 106) specimens analysed (**Table 2**). *Anopheles* specimens analyzed (N = 15) for *kdr* mutations at the *Vgsc* locus 1014 were homozygous for the wild type susceptible allele L1014 (100%).

## Discussion

Crete is one of Greece’s leading tourist destinations receiving over 5.0 million tourists in 2019 alone [59]. Tourism is one of the most important industries on the island and a major contributor to employment (in 2019 the local tourist industry numbered 39,900 employees) [60]. However, this vital element of local and national economy faces a potential risk in the establishment and emergence, /re-emergence of mosquito-borne disease. In support of designing and implementing evidence based vector control actions in Crete, a three-year integrative surveillance program targeting mosquitoes and VBD pathogens was established across the island in 2018.

During the surveillance program, important vector species were recorded in all four prefectures including the major WNV vector *Cx*. *pipiens ss* [61], *Ae*. *albopictus–*vector of over 22 arboviruses and the malaria vector *An*. *superpictus*.

*Cx*. *pipiens* s.s. was by far the most dominant species across the island, representing >85% of the collected specimen within each prefecture between 2018–2020. These results are similar to the relative abundance of *Cx*. *pipiens* recorded from Crete in 2014–16 (75–97%) [14], demonstrating a steady state population dominance for the vector species (in spite of any small seasonal abundance fluctuations between 2014–2020). Throughout the surveillance program the highest *Cx*. *pipiens* numbers were consistently recorded in the western prefectures (Chania and Rethymnon), peaking in 2018. These population dynamics may partially be explained by the precipitation patterns occurring in Crete which increased in 2018 compared to 2019 and 2020, with most 2018–20 rain events occurring in Chania-Rethymnon (compared to Heraklion-Lasithi) [62]. The observed seasonality of *Cx*. *pipiens* populations is in accordance with available literature from other regions of Greece, with June-September being the period with maximum activity [6].

Overall, under a comparative prism the number of mosquitoes collected in Crete (an island with distinct ecological characteristics and climatic conditions) appears minimal compared to the mosquito numbers recorded in other regions of Greece, reflecting the mosquito reproduction capacity of the island. Indicatively in Crete, in 2018 (i.e., the year where population numbers were highest) an average of 18.3 female mosquitoes were collected per CDC light trap (baited with dry ice) per night compared to an average of 1944 female mosquitoes collected per CDC light trap (baited with dry ice) per night in the region of Thessaloniki in 2014 [6]. In any case, the collection of vector populations within urban, semi-urban and rural settings in Crete and the dominant presence of vector species in all four prefectures is of high epidemiological relevance and highlights the need for ongoing surveillance activities.

Notably, the prepotent abundance of *Cx*. *pipiens* across Crete, in conjunction with the representation of *Cx*. *pipiens* hybrids (>20%), in Chania, Rethymnon, and Heraklion comprises a situational risk factor for WNV transmission, which should not be overlooked.

The occurrence of *Ae*. *albopictus* in all four prefectures is in line with previous entomological findings [9,14] while the vector’s possible dominant presence over the native non vector *Ae*. *cretinus* possibly indicates an antagonistic displacement at play. It is possible that the recorded species abundance is an underestimation of the vector’s true numbers as *Ae*. *albopictus* is a day biting mosquito with bimodal biting peaks at dawn and in the afternoon whilst the CDC light–CO2 traps were set from 18.00–08.00, hence only capturing the vector’s peak activity [63].

Three *Anopheles* species were recorded in Crete: *An*. *superpictus* which is considered a major malaria vector in the middle East and central Asia [64]; and the secondary malaria vectors *An*. *claviger* and *An*. *algeriensis*. Although few *Anopheles* specimen were collected (potentially due to the sole deployment of CDC light traps baited with dry ice [65], indicating the need for deploying robust anopheline trapping measures) the aforementioned species’ occurrence and the possible presence of other malaria vectors currently going undetected indicates potential risk for malaria transmission. Future active surveillance with a combination of mosquito collection methods (e.g. larval collections, light traps, ovitraps, BG sentinel traps, human landing catches and others), in accordance to the surveillance aims and entomological context [65,66], will enhance further the generation of information on the presence, distribution, abundance and seasonal activity of the native and invasive mosquito fauna.

*Cx*. *pipiens*, *Ae*. *albopictus*, and sentinel chickens were systematically monitored for flavivirus/WNV infections. Although no mosquito pools were found positive, WNV specific antibodies were detected in chicken blood samples collected from Rethymnon in 2018 corresponding to a 1.7% detection rate (for 2018) indicating the circulation of WNV amongst local vector populations and bird hosts (albeit at low levels). This finding is in line with the occurrence of two human WNV cases in Rethymnon in the same year [25]. Particularly, the detection of WNV antibodies in chicken sera preceded the (2^nd^) human WNV case (i.e., symptom onset) by approximately two weeks (the 1^st^ human case was recorded 1 month prior program initiation) pinpointing the importance of active surveillance as an integral component of early warning systems for WNV [67], informing clinicians and public health stakeholders to take appropriate prevention/control actions while guiding targeted and timely vector control interventions. The validity of this approach has been described in several studies including [21] whom detected circulation of WNV in sentinel chickens 1 month prior the onset of the first human cases in Northern Greece in 2011 and [68] where WNV seropositive chickens preceded the first human cases by over 6 weeks in Florida, 2001.

The CHS mutation I1043F, shown to confer high DFB resistance, was detected at an 8% allelic frequency in *Cx*. *pipiens* collected from Agios Nikolaos locality in Lasithi (2020). Markedly, this is the first report of a CHS resistance mutation in *Culex* collected from Greece despite extensive screening in the past (in 2014–2017 field collected samples) [7,35,36] and the first documented occurrence of homozygous 1043F following I1043L/M/F detection in populations from northern Italy [34–36], southern France [36], and western Turkey [37]. The occurrence of I1043F in Cretan *Cx*. *pipiens* mosquitoes, whether signifying a novel I1043F emergence event or attributed to the migration of specimens harbouring the resistant allele, should not be left un-addressed. The potential selection for the resistance trait fuelled by agricultural and/or mosquito control DF based applications may seriously impede the effectiveness of DFB. CHS screening in *Ae*. *albopictus*, recorded only the wild type allele I1043, suggesting the current suitability of DFB for local *Ae*. *albopictus* control.

High *kdr* mutation frequencies were recorded from populations of *Cx*. *pipiens* across the island. The cumulative L1014F/C/S mutation frequencies in each prefecture (>92%) were higher in comparison to the *kdr* frequencies recorded in other populations from Greece (ranging from 28–88.3%) [6,7,43] while mutation L1014C was the most commonly found allele in the Cretan populations (≥74%). Few studies have reported the presence of the L1014C mutation in *Culex* mosquitoes worldwide [69], including a study from Greece [6] reporting L1014C presence in *Culex* populations from northern Greece (>90%) and [70] whom reported the mutation in *Cx*. *pipiens* populations from western Turkey at an allele frequency of 53%.

To our knowledge, the exact contribution/effect of L1014C towards pyrethroid resistant phenotypes remains elusive. However functional characterization of L1014C through the expression of mutated VGSCs in *Xenopus* eggs (coupled with electrophysiological property analyses) showed the mutation to confer a slight reduction (≤4.2-fold) in VGSC sensitivity to permethrin and deltamethrin [40]. Despite the putative low operational relevance of L1014C the mutation’s high frequency alongside the occurrence of L1014F highlights the need for systematic *kdr* mutation monitoring in local populations.

Mutation L1014S, recorded at low frequencies in populations from Chania, Rethymnon, and Heraklion to our knowledge has previously only been reported in *Cx*. *pipiens* complex mosquitoes form China, Japan and USA [71,72]. Admittedly, the exact distribution and frequency of L1014C and L1014S in *Culex* field populations remains largely unknown and is possibly underestimated as no multiplex molecular diagnostic assay is currently available for simultaneously detecting and differentiating the presence of the three resistance alleles while the classical molecular assay used in many resistance studies appears to not distinguish L1014C from the widely distributed L1014F.

*Ae*. *albopictus* harbored *kdr* mutations I1532T and F1534C at a frequency range of (2.3–8.7%) and (25–46.6%) respectively, but not mutation V1016G associated with stronger pyrethroid resistant phenotypes [41,73]. Although F1534C has been associated with permethrin resistance in the homozygous state [74], the mutation was recorded predominantly in heterozygosis conferring non-significant resistance levels due to the mutated allele’s recessive nature. Our findings are in unison with a recent study including *Ae*. *albopictus* mosquitoes from Crete, which reported the non-detection of V1016G and similar mutation frequencies ranging from (1.7–6.5%) for I1532T and (29–48.3%) for F1534C [9]. In *Anopheles* no *kdr* mutations were recorded, as was the case in screened samples from northern Greece and the island of Chios [6,7]. The close to fixation *kdr* mutation frequencies recorded in *Cx*. *pipiens* in Crete, and the occurrence of *kdr* mutations in *Ae*. *albopictus* populations across the island could reflect the passive transportation of mosquitoes harbouring resistance mutations and/or the exposure of mosquito populations to local insecticidal pressures.

To date no large scale pyrethroid based interventions targeting mosquitoes have taken place in Crete, yet pyrethroids are extensively used against agricultural pests [75]. Specifically, the pyrethroids: lambda-cyhalothrin, beta-cyfluthrin, deltamethrin and alpha-cypermethrin, have been widely used over the last decade against the olive fruit fly *Bactrocera oleae*, with a recent study showing a 22-fold pyrethroid resistance level increase in field *B*. *oleae* populations compared to susceptible strains) attributed to the up regulation of specific P450s [32]. It is possible that this strong pyrethroid pressure may have also selected for the high *kdr* mutation frequencies recorded in the *Cx*. *pipiens* mosquitoes and the respective mutation frequencies in *Ae*. *albopictus*. This is in consonance with a number of studies where pyrethroid, diflubenzuron, and neonicotinoid resistance in mosquitoes was correlated with extensive agricultural insecticide applications of the corresponding active ingredients [6,35,76].

Although OPs are only rarely used for vector control and agricultural purposes in the EU, *Ace-1 –*based target site resistance and CCEs-based metabolic resistance has been recorded in *Cx*. *pipiens* and *Ae*. *albopictus* populations from Greece, respectively [6,9,43]. Inclusion of these markers in systematic monitoring programs is important, in view of possible re-introduction of OPs and carbamates for vector control, such as pyrimiphos-methyl and bendiocarb IRS formulations currently available, in case of emergency situations [77].

Formulations of *Bti* are a valuable alternative group of public health insecticides against which no resistance has been documented to date in any mosquito species. In Crete, vector control reliance on *Bti* is minimal due to the current efficacy of DFB against local mosquito population and the limited presence of large water bodies/immature development sites [78]. However, *Bti* based products are used to an extent for agricultural pest control purposes. In the absence of any molecular markers for monitoring incipient *Bti* resistance targeted larval collections will enable the realization of *Bti* bioassays.

In view of retaining the current DFB efficacy and safeguarding pyrethroid product effectiveness for mosquito control purposes, systematic monitoring of CHS1 locus 1043 in local *Cx*. *pipiens* populations, investigation of L1014C operational significance upon homozygosity, development of novel diagnostic tools discriminating the three VGSC 1014 *kdr* mutations (1014F/C/S) in *Culex* mosquitoes and evaluation of *Bti* resistance in local vector populations, may significantly contribute towards the development of appropriate insecticide resistance management programs.

In conclusion, the occurrence of important disease vectors across the island of the Crete in conjunction with the detection of WNV positive chickens (in 2018) and DFB, pyrethroid resistance mutations in vector populations, highlight the need for establishing: (i) robust entomological, insecticide resistance, and pathogen surveillance systems in support of VBD evidence-based control and (ii) appropriate insecticide resistance management programs ensuring the efficacy and sustainable use of DFB and pyrethroid based products in vector control. In light of protecting public health and safeguarding the tourist economy in Greece, the development and utilization of information technology tools (e.g. [79]) providing decision support through driving and facilitating the collection, analysis and interpretation of surveillance data and its transformation into actionable information will help establish direct links between vector/pathogen surveillance programs and vector/disease control efforts resulting in optimum VBD control outcomes.

## Supporting information

S1 Table Molecular assays used in the study. (DOCX)

S2 Table Primers and probes used in this study for regular and quantitative real-time PCR. (DOCX)

S3 Table Number of *Cx*.*pipiens* and *Ae*. *albopictus* pools and specimens analysed for flavivirus and WNV. (DOCX)

S1 Fig Application of pan-Flavivirus assay in mosquito pools with regular PCR (A), West Nile virus lineage 1/lineage 2 assay with multiplex TaqMan qRT-PCR (B) and r18S internal control (IC) assay (B) in mosquito pools from Crete. (DOCX)
