# Supplementary material for: Mosquito population structure, pathogen surveillance and insecticide resistance monitoring in urban regions of Crete, Greece
Source: PLoS Negl Trop Dis. 2022 Feb 17;16(2):e0010186. doi: 10.1371/journal.pntd.0010186 (PMC8890720; doi:10.1371/journal.pntd.0010186)
Supplement: S1 Table — (DOCX) [file pntd.0010186.s001.docx]

**S1 Table** Molecular assays used in the study

|  | **Species** | **Assay** | **Primers/Probes*** | **PCR reaction** |  | **Thermal protocol** | **% w/v agarose gel** | **Product size** |
| --- | --- | --- | --- | --- | --- | --- | --- | --- |
| Molecular identification of mosquito species | *Culex pipiens* complex | Diagnostic PCR for species identification (*ace*-2) | ACEquin, ACEpal, ACEpip, ACEtorr, B1246s | 10-20 ng gDNA  0.5 μM of each primer  0.4 μM dNTPs  1X KAPA *Taq* Buffer A  2 mM MgCl_2_  0.15 mg/ml BSA  1 U of KAPA *Taq* pol  Reaction volume: 20 μl |  | 95°C 5 min, 40 cycles ×  (95°C 30 s, 55°C 30 s,  72°C 1 min), 72°C 5 min | 2.0 | 610 bp: *Cx. pipiens*  274 bp: *Cx. quinquefasciatus*  416 bp: *Cx. torrentium*  478 bp: *Cx. p. pallens* |
|  | *Cx. pipiens* | Diagnostic PCR for biotype identification (CQ11) | PipR, MolR, BioComF | 10-20 ng gDNA  0.3-0.4 μM of each primer  0.4 μM dNTPs  1X Ampli *Taq* Buffer  1.5- 2 mM MgCl_2_  1.5 U of Ampli *Taq* pol  Reaction volume: 20 μl |  | 95°C 5 min, 40 cycles ×  (95°C 30 s, 54°C 30 s,  72°C 40 s), 72°C 5 min | 2.0 | 200 bp: *Cx. p. pipiens*  250 bp: *Cx. p. molestus*  200 bp + 250 bp: hybrid |
|  | *Aedes* spp. | Diagnostic PCR for species identification (ITS2) | 5.8S, 28S | 10-20 ng gDNA  0.3-0.4 μM of each primer  0.4 μM dNTPs  1X KAPA *Taq* Buffer A  1.5- 2 mM MgCl_2_  1.5 U of KAPA *Taq* pol  Reaction volume: 25 μl |  | 95°C 5 min, 40 cycles ×  (95°C 30 s, 52°C 30 s,  72°C 30 s), 72°C 5 min | 1.5 | 508 bp: *Ae. albopictus*  385 bp: *Ae. cretinus* |
|  | *Anopheles* spp. | Species identification (COI)- Sequencing | C1-J-1718,  C1-N-2191 |  |  | 95°C 2 min, 40 cycles ×  (95°C 30 s, 50°C 30 s,  72°C 50 s), 72°C 5 min | 1.0 | 522 bp |
|  | *Anopheles* spp. | Species identification (ITS2)- Sequencing | 5.8S, 28S |  |  | 94 °C 2 min, 40 cycles × (94 °C 30 s, 53 °C 30 s, 72 °C 50 s), 72 °C 10 min | 1.5 | 300-700 bp |
| Monitoring of target-site resistance mutations | *Cx. pipiens* | *VGSC* L1014F/C/S- Sequencing | Cx1014F, CgD2 | 10-20 ng gDNA  0.3-0.4 μM of each primer  0.4 μM dNTPs  1X KAPA *Taq* Buffer A  1.5- 2 mM MgCl_2_  1.5 U of KAPA *Taq* pol  Reaction volume: 25 μl |  | 95°C 2 min, 40 cycles ×  (95°C 30 s, 54°C 30 s,  72°C 30 s), 72°C 5 min | 1.5 | 476 bp |
|  | *Ae. albopictus* | *VGSC* V1016G-  Sequencing | Kdr2F, Kdr2R |  |  | 95°C 5 min, 40 cycles ×  (95°C 30 s, 55°C 30 s,  72°C 30 s), 72°C 5 min | 1.5 | 500 bp |
|  | *Ae. albopictus* | *VGSC* I1532T, F1534C/L/S –  Sequencing | AegSCF7, AegSCR7 |  |  | 95°C 5 min, 40 cycles ×  (95°C 30 s, 57°C 30 s,  72°C 1 min), 72°C 10 min | 1.5 | 740 bp |
|  | *Anopheles* spp. | *VGSC* L1014F/C/S-  Sequencing | AnHF, AnHR |  |  | 95°C 5 min, 35 cycles ×  (95°C 30 sec, 52°C 30 sec, 72°C 40 sec), 72°C 5 min | 1.5 | 207 bp |
|  | *Cx. pipiens* | *CHS1* I1043L/M/F- Sequencing | Kkv F1, Kkv R2 |  |  | 95°C 5 min, 40 cycles ×  (95°C 30 s, 61°C 30 s,  72°C 1 min), 72°C 5 min | 1.5 | 820 bp |
|  | *Ae. albopictus* | *CHS1* I1043L/M/F- Sequencing | Kkv F3, Kkv R3 |  |  | 95°C 5 min, 40 cycles ×  (95°C 30 s, 55°C 30 s,  72°C 1 min), 72°C 5 min | 1.5 | 340 bp |
| Pathogen detection | *Cx. pipiens*  *Aedes* spp. | Pan-Flavivirus detection | Plan-Fl_F  Plan-Fl_R | 100 ng total RNA  1.0 μM of each primer and 0.3 μM of each probe  RT-PCR master mix (FTD, Luxembourg)  Reaction volume: 10 μl |  | 50 °C–30 min, 95 °C–3 min, 40 cycles×[95 °C–15 s, 55 °C–20 s, 72 °C–60 s], 72 °C–10 min. | 1.5 | 266 bp |
|  | *Cx. pipiens*  *Aedes* spp. | WNV (lineage 1 vs lineage 2) | WNV_F  WNV_R  WNV_P1  WNV_P2 | 100 ng total RNA  0.5 μM of each primer and 0.3 μM of each probe  RT-PCR master mix (FTD, Luxembourg)  Reaction volume: 10 μl |  | 50 °C–15 min, 95 °C–3 min, and 45 cycles× [95 °C–3 s and 60 °C−30 s]. | N/A | 76 bp |
|  | *Cx. pipiens* | Internal mosquito control targeting r18S | r18S_F  r18S_R | 100 ng total RNA  0.3 μM of each primer  RT-PCR master mix (FTD, Luxembourg)  SYBR green safe 20x  Reaction volume: 10 μl |  | 50 °C–15 min, 95 °C–3 min, and 45 cycles× [95 °C–3 s and 60 °C−30 s],  Melting curve protocol. | N/A | 159 bp |

***Ace-2*:** Acetylcholinesterase-2; **CQ11**: Microsatellite locus; **ITS2**: Internal transcribed spacer two; **COI**: Cytochrome oxidase subunit 1; **VGSC**: Voltage gated sodium channel; **CHS1**: Chitin synthase; ***** All primer sequences are provided in S1 Table
