# Supplementary material for: Mosquito population structure, pathogen surveillance and insecticide resistance monitoring in urban regions of Crete, Greece
Source: PLoS Negl Trop Dis. 2022 Feb 17;16(2):e0010186. doi: 10.1371/journal.pntd.0010186 (PMC8890720; doi:10.1371/journal.pntd.0010186)
Supplement: S2 Table — (DOCX) [file pntd.0010186.s002.docx]

**S2 Table** Primers and probes used in this study for regular and quantitative real-time PCR

| **Primers/Probes** | **Sequence (5’ → 3’)** |
| --- | --- |
| ACEpip | GGAAACAACGACGTATGTACT |
| ACEquin | CCTTCTTGAATGGCTGTGGCA |
| ACEpal | ATGGTGGAGACGCATGACG |
| ACEtorr | TGCCTGTGCTACCAGTGATGTT |
| B1246s | TGGAGCCTCCTCTTCACGG |
| PipR | CATGTTGAGCTTCGGTGAA |
| MolR | CCCTCCAGTAAGGTATCAAC |
| BioComF | GATCCTAGCAAGCGAGAAC |
| 5.8S | TGTGAACTGCAGGACACATG |
| 28S | ATGCTTAAATTTAGGGGGTA |
| C1-J-1718 | GGAGGATTTGGAAATTGATTAGTTC |
| C1-N-2191 | CCCGGTAAAATTAAAATATAAACTTC |
| Cx1014F | TGATTGTGTTCCGGGTGCTG |
| CgD2 | GCAAGGCTAAGAAAAGGTTAAG |
| Kdr 2F | TTCACCGACTTCATGCACTC |
| Kdr 2R | CGCAATCTGGCTTGTTAACTT |
| AegSCF7 | GAGAACTCGCCGATGAACTT |
| AegSCR7 | GACGACGAAATCGAACAGGT |
| AegSCR8 | AGCTTTCAGCGGCTTCTTC |
| AnHF | TGGATTGAATCAATGTGGGATTC |
| AnHR | AAGGATAAGAACCGAAATTGGAC |
| Kkv F1 | CCGCGTTCAAGATTGACAACTGG |
| Kkv F2 | GGCGAGGACGGAATCGG |
| Kkv R2 | TCCAGTAGGGGTTCGTCAGG |
| Kkv R3 | TGGATACTTCAATGGAACCTTCC |
| Kkv F3 | TCGGAAGTCCTTCGGCTTATTC |
| Plan-Fl_F | TACAACATGATGGGAAAGAGAGAGAA |
| Plan-Fl_R | GTGTCCCAGCCGGCGGTGTCATCAGC |
| WNV_F | GTGATCCATGTAAGCCCTCAGAA |
| WNV_R | GTCTGACATTGGGCTTTGAAGTTA |
| WNV_P1 | FAM-AGGACCCCACATGTT-MGB |
| WNV_P2 | HEX-AGGACCCCACGTGCT-MGB |
| r18S_F | CGCGGTAATTCCAGCTCCACTA |
| r18S_R | GCATCAAGCGCCACCATATAGG |
