# Supplementary material for: Mosquito population structure, pathogen surveillance and insecticide resistance monitoring in urban regions of Crete, Greece
Source: PLoS Negl Trop Dis. 2022 Feb 17;16(2):e0010186. doi: 10.1371/journal.pntd.0010186 (PMC8890720; doi:10.1371/journal.pntd.0010186)
Supplement: S3 Table — (DOCX) [file pntd.0010186.s003.docx]

**S3 Table** Number of *Cx.pipiens* and *Ae. albopictus* pools and specimens analysed for flavivirus and WNV

|  | **No of positive/tested pools (No of mosquitoes) per prefecture - species - year** | | | | | |
| --- | --- | --- | --- | --- | --- | --- |
|  | *Culex pipiens* | | | *Aedes albopictus* | | |
|  | 2018 | 2019 | 2020 | 2018 | 2019 | 2020 |
| **Prefecture** |  |  |  |  |  |  |
| Chania | 0/0 | 0/39 (489) | 0/26 (279) | 0/0 | 0/2 (13) | 0/1 (10) |
| Rethymnon | 0/40 (706) | 0/57 (668) | 0/23 (254) | 0/4 (86) | 0/6 (56) | 0/5 (25) |
| Heraklion | 0/16 (325) | 0/13 (109) | 0/10 (97) | 0/0 | 0/0 | 0/0 |
| Lasithi | 0/0 | 0/8 (62) | 0/7 (42) | 0/0 | 0/0 | 0/0 |
