# Supplementary material for: Mosquito population structure, pathogen surveillance and insecticide resistance monitoring in urban regions of Crete, Greece
Source: PLoS Negl Trop Dis. 2022 Feb 17;16(2):e0010186. doi: 10.1371/journal.pntd.0010186 (PMC8890720; doi:10.1371/journal.pntd.0010186)
Supplement: S1 Fig — (DOCX) [file pntd.0010186.s004.docx]

**S1 Figure:** Application of pan-Flavivirus assay in mosquito pools with regular PCR (A), West Nile virus lineage 1/lineage 2 assay with multiplex TaqMan qRT-PCR (B) and r18S internal control (IC) assay (B) in mosquito pools from Crete. All samples tested were found negative both for flaviviruses and WNV. The positive signal obtained from the IC demonstrates successful amplification, thereby validating a negative result for the primary target (WNV). PC: positive control, NC: negative control, M: Molecular weight DNA marker, RFU: Relative Fluorescence Units.
